# SARS-Like Coronavirus WIV1-CoV Does Not Replicate in Egyptian Fruit Bats (*Rousettus aegyptiacus*)

**DOI:** 10.3390/v10120727

**Published:** 2018-12-19

**Authors:** Neeltje van Doremalen, Alexandra Schäfer, Vineet D. Menachery, Michael Letko, Trenton Bushmaker, Robert J. Fischer, Dania M. Figueroa, Patrick W. Hanley, Greg Saturday, Ralph S. Baric, Vincent J. Munster

**Affiliations:** 1 Laboratory of Virology, National Institute of Allergy and Infectious Diseases, National Institutes of Health, Hamilton, MT 59840, USA; neeltje.vandoremalen@nih.gov (N.v.D.); michael.letko@nih.gov (M.L.); bushmakertj@niaid.nih.gov (T.B.); fischerro@niaid.nih.gov (R.J.F.); dania.figueroa@nih.gov (D.M.F.); 2 Department of Epidemiology, University of North Carolina at Chapel Hill, Chapel Hill, NC 27599, USA; aschaefe@email.unc.edu (A.S.); vimenach@UTMB.EDU (V.D.M.); rbaric@email.unc.edu (R.S.B.); 3 Rocky Mountain Veterinary Branch, National Institute of Allergy and Infectious Diseases, National Institutes of Health, Hamilton, MT 59840, USA; patrick.hanley@nih.gov (P.W.H.); greg.saturday@nih.gov (G.S.)

**Keywords:** WIV1-CoV, coronavirus, emerging infectious diseases, animal model

## Abstract

Severe acute respiratory syndrome (SARS)-like WIV1-coronavirus (CoV) was first isolated from *Rhinolophus sinicus* bats and can use the human angiotensin converting enzyme 2 (ACE2) receptor. In the current study, we investigate the ability of WIV1-CoV to infect *Rousettus aegyptiacus* bats. No clinical signs were observed throughout the experiment. Furthermore, only four oropharyngeal swabs and two respiratory tissues, isolated on day 3 post inoculation, were found positive for viral RNA. Two out of twelve bats showed a modest increase in coronavirus specific antibodies post challenge. In conclusion, WIV1-CoV was unable to cause a robust infection in *Rousettus aegyptiacus* bats.

## 1. Introduction

Emerging infectious diseases form a significant threat to the human population. The natural history of viruses in animal reservoirs and their ability to infect the human host is important to predict the risk of spillover events and subsequent epidemics. The World Health Organization has identified a list of top emerging diseases likely to cause major epidemics. The list includes severe acute respiratory syndrome coronavirus (SARS-CoV), which caused a pandemic in 2002–2003 [1]. SARS-CoV is hypothesized to originate from *Rhinolophus sinicus* bats since several SARS-like viruses have been detected in this host [2,3]. However, most SARS-like viruses from bats do not bind the human receptor, angiotensin-converting enzyme 2 (ACE2). For example, a recombinant virus based on a consensus sequence of SARS-like viruses from bats could not be cultured unless a part of the spike protein was exchanged with SARS-CoV spike protein [4]. This suggests that a substantial portion of the SARS-like viruses circulating in bats cannot infect humans directly.

In 2013, Ge et al. isolated a SARS-like virus from the Chinese horseshoe bat (*Rhinolophus sinicus*): WIV1-CoV [5], which can use human angiotensin-converting enzyme 2 (ACE2), the receptor for SARS-CoV, in vitro and transgenic mice [6]. This suggests WIV1-CoV may be able to directly transmit from bats to humans. In order to understand the relationship between coronaviruses and their bat hosts, experimental animal models are needed. Unfortunately, most bat species are difficult to obtain for laboratory experiments and most of these species have poorly annotated genomes. These limitations have posed a challenge in the field and explain a current deficit in the literature. In 2010, one group inoculated Leschenault’s rousette bats (*Rousettus leschenaultia*) with a homogenate of Betacoronavirus (clade b)-positive intestinal tissues obtained from a lesser short-nosed fruit bat (*Cynopterus brachyotis*). No clinical signs were observed, but viral RNA was detected in both fecal samples and intestinal tissue [7]. Likewise, no clinical signs were observed following intranasal and intraperitoneal inoculation of Jamaican fruit bats (*Artibeus jamaicensis*) with Middle East respiratory syndrome (MERS)-CoV. However, shedding of viral RNA was detected in oral and rectal swabs, and a variety of different tissues tested positive for viral RNA [8]. Together, these studies suggest that bats might show limited clinical signs when infected with coronaviruses but will continue to shed the virus.

In this study, we investigate the ability of WIV1-CoV to infect and replicate in *Rousettus aegyptiacus* bats.

## 2. Materials and Methods

### 2.1. Ethics Statement

Animal experiments were approved by the Institutional Animal Care and Use Committee of the Rocky Mountain Laboratories (ASP 2016-021E, 05/2016). Experiments were performed following all guidelines and basic principles of the United States Public Health Service Policy on Humane Care and Use of Laboratory Animals and the Guide for the Care and Use of Laboratory Animals. Experiments with infectious WIV1-CoV under BSL3 conditions was approved by the Institutional Biosafety Committee (IBC). IBC-approved standard operating procedures were followed for inactivation and removal of samples from high containment.

### 2.2. Virus and Cells

An infectious clone of WIV1-CoV was generated as previously described [6]. Virus propagation was performed in VeroE6 cells in DMEM (Sigma, St. Louis, MO, USA) supplemented with 2% fetal bovine serum (Gibco, Grand Island, NY, USA), 1 mM l-glutamine (Lonza), 50 U/mL penicillin, and 50 μg/mL streptomycin (Gibco, Grand Island, NY, USA) (2% DMEM). VeroE6 cells and baby hamster kidney (BHK) cells were maintained in DMEM supplemented with 10% fetal bovine serum, 1 mM l-glutamine, 50 U/mL penicillin, and 50 μg/mL streptomycin (10% DMEM). The virus was titrated by inoculating VeroE6 cells with tenfold serial dilutions of virus in 2% DMEM. Five days after inoculation, cytopathic effect (CPE) was scored and TCID_50_ was calculated from four replicates using the method of Spearman-Karber.

### 2.3. Spike Incorporation into VSV Reporter Particles

Equivalent volumes of VSV reporter particles encoding luciferase and WIV1-spike, MERS-CoV spike or SARS-CoV spike were concentrated over an OptiPrep cushion (10% in PBS; Sigma, St. Louis, MO, USA) at 20,000× *g* for 2 h at 4 °C. Particle pellets were resuspended in lysis buffer (1% SDS, 1× ThermoFisher NuPage LDS (Waltham, MA, USA), 1× ThermoFisher NuPage DTT), boiled for 10 min at 100 °C and analyzed for FLAG expression on a 10% Bis-Tris PAGE gel (NuPage; Thermofisher).

### 2.4. VSV Pseudotype Entry Assay

Pseudotyped luciferase VSV reporter particles were produced as previously described [9]. ACE2-transfected BHK cells were then infected with equal volumes of VSV reporter particles pseudotyped with MERS-CoV, SARS-CoV, or WIV1-CoV spike.

### 2.5. ACE2-Dependent Replication Kinetics of WIV1

BHK cells were transfected in 6-well plates with 4 μg pcDNA3.1(+) containing ACE2 from *Rousettus aegyptiacus* (XM016118926) or *homo sapiens* (AB193259) using 7.5 μL of Lipofectamine 3000 and 8 µL 3000 reagent (Thermo Fisher, Waltham, MA, USA). Replication kinetics were determined by inoculating cells with a multiplicity of infection (MOI) of 0.01 WIV1-CoV in triplicate 24 h post-transfection. One hour after inoculation, cells were washed twice with PBS, and fresh media was placed on the cells. Supernatants were sampled at 0 and 48 h after inoculation. Virus titers in supernatants were determined as described.

### 2.6. Animal Experiment

Twelve male adult Egyptian fruit bats (*Rousettus aegyptiacus*) were obtained from the Catoctin Wildlife Preserve and Zoo in Thurmont, Maryland. All bats were inoculated with 10^5^ TCID_50_ of WIV1-CoV via the intranasal (25 µL per nare), intratracheal (100 µL) and intra-esophageal route (100 µL). Exams were performed daily and included measuring body weight and temperature, obtaining oropharyngeal, rectal and urogenital swabs, and scoring for clinical signs of disease. On day 3, 7, and 28, four animals were euthanized and nasal turbinates, larynx, pharynx, trachea, lung, brain, eye, conjunctiva, heart, liver, spleen, kidney, bladder, reproductive organs, stomach, proximal and distal intestinal tract, cervical lymph nodes, adrenal glands, skin, and skeletal muscle were collected for virological and histopathological analyses. Lung weights were measured upon necropsy.

### 2.7. Viral RNA Detection

Tissues were homogenized in RLT buffer, and RNA was extracted using the RNeasy method on the QIAxtractor (Qiagen, Venlo, The Netherlands) according to the manufacturer’s instructions. RNA was extracted from swab samples using the QiaAmp Viral RNA kit on the QIAxtractor. For one-step real-time qPCR, 5 μL RNA was used in the QuantiFast Probe PCR kit (Qiagen), according to instructions by the manufacturer. Standard dilutions of a virus stock with known titer were run in parallel in each run, to calculate TCID_50_ equivalents in the samples. Primers and probe were designed for detection of WIV1-CoV (Forward primer: TCAGGCTGGAAATGCTACAG, reverse primer: GTCCTCCACTTGCTAGGTAATC, probe: TGTGCTTTCCTTCTGTGCCTTTGC). Primers and probes specific for *Rousettus aegyptiacus* GAPDH and HPRT were designed as extraction controls (GAPDH: forward primer: GGTTGTCTCCTGCGACTTTA, reverse primer: CCTGTTGCTGTAGCCAAAT TC, probe: AAAGTGGTCATTGAGGGCAATGCC. HPRT forward primer: AGATGGTGAAGGTCGCAAG, reverse primer: CCTGAAGTATTCATTATAGTCAAGGG, probe: ACTTTGTTGGATTTGAAATTCCAGACA AGTTTG. All qRT-PCR cycles were as follows: 15 min at 50 °C, 5 min at 95 °C, then 40 cycles of 15 s at 95 °C and 15 s (WIV1) or 30 s (Glyceraldehyde 3-phosphate dehydrogenase (GAPDH) and Hypoxanthine-guanine phosphoribosyltransferase (HPRT)) at 60 °C.

### 2.8. Hematology

EDTA blood was obtained from bats pre-challenge (D-95 and D-2) and day of necropsy. The total white blood cell, lymphocyte, neutrophil, monocyte, eosinophil, and basophil count were determined with the IDEXX ProCyte DX analyzer (IDEXX Laboratories, Westbrook, ME, USA).

### 2.9. Histopathology

Tissues were fixed in 10% neutral buffered formalin for a minimum of seven days. Sagittal-cut skulls were decalcified in 20% EDTA in sucrose (Newcomer Supply, Middleton, WI, USA), changed weekly for six weeks. Tissues were then processed using a Sakura VIP-6 Tissue Tek tissue processor and embedded in Ultraffin paraffin polymer (Cancer Diagnostics, Durham, NC, USA). Samples were sectioned at 5 µm, and resulting slides were stained with hematoxylin and eosin. For WIV1-CoV immunohistochemistry (IHC), tissues were stained with SARS NP rabbit polyclonal antibody (Novus Biologicals, NB100-56576, 1:250, Centennial, CO, USA). For ACE2 IHC, tissues were stained with ACE2 rabbit polyclonal antibody (Abcam, ab15348, 1:1500). Hereafter, antibodies were detected using ImmPress VR Polymer HRP anti-rabbit IgG (Vector Laboratories, MP-6401-15, no dilution, Burlingame, CA, USA) followed by Discovery ChromoMap DAB kit (Ventana Medical Systems, 760-159, Tucson, AZ, USA).

### 2.10. Serology and Microneutralization

Enzyme-linked immunosorbent assay (ELISA) was performed by coating SARS-CoV spike S1 or nucleoprotein (N) antigen (NR686 and NR48761, BEI resources, 0.5 µg/mL in 50 mM bicarbonate binding buffer (4.41 g KHCO_3_ and 0.75 g Na_2_CO_3_ in 1 L water)) on to MaxiSorb plates (Nunc, Rochester, NY, USA). Plates were blocked with 5% non-fat dried milk in PBS-0.1% Tween (5MPT). Serum samples were diluted in 5MPT and incubated on plates for 1 h. Detection of antibodies was performed with HRP-conjugated IgG (H+L) secondary antibody and developing solution (Seracare, Milford, KS, USA) followed by a measurement at 450 nm. Sera was termed seropositive if the optical density (OD) value was higher than 0.2. To determine microneutralization titers, heat-inactivated sera (30 min at 56 °C) was diluted in 2% DMEM, then incubated 1 h at 37 °C and 5% CO_2_ with 100 TCID_50_ of WIV1-CoV. Serum:virus mixture was added to VeroE6 cells and five days after inoculation, CPE was scored. Neutralizing titer was determined from two replicates.

### 2.11. Statistical Analysis

Statistical analysis was performed using Student’s *t*-test on Graphpad Prism 7 for Windows. *p*-values < 0.05 were considered as significant.

## 3. Results

**Figure 1:**
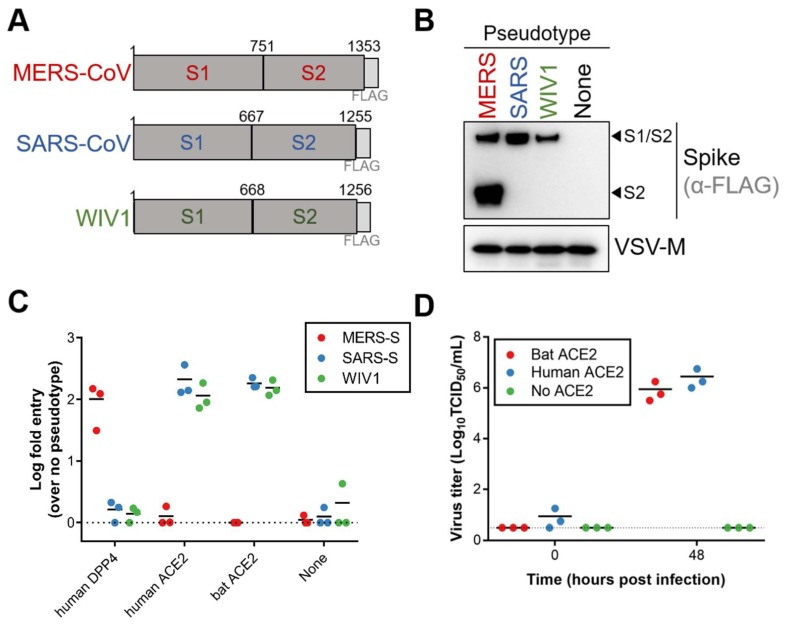
Egyptian fruit bat ACE2 is a suitable receptor for WIV1-CoV. (**A**) Schematic overview of coronavirus spike proteins. (**B**) VSV particles pseudotyped with coronavirus spike proteins were concentrated and analyzed for spike incorporation by Western blot. (**C**) BHK cells were transfected with coronavirus receptors and infected with pseudotyped particles in triplicate. (**D**) BHK cells were transfected with ACE2 plasmids and inoculated with WIV1-CoV at a MOI of 0.01, 24 h after transfection. Supernatants were harvested at 0, and 48 hpi and viral titers were determined by endpoint titration in quadruplicate in VeroE6 cells.

Because variation in ACE2 has been shown to act as a host species barrier [10], we first tested if WIV1 spike could interact with Egyptian fruit bat ACE2. Single-cycle VSV luciferase reporter particles were generated with FLAG-tagged MERS-CoV, SARS-CoV or WIV1 spike (Figure 1A). All three coronavirus spike proteins incorporated into VSV reporter particles (Figure 1B). While MERS-CoV spike could only mediate entry into cells expressing DPP4, both SARS-CoV and WIV1 spikes mediated entry into cells expressing human and Egyptian fruit bat ACE2 (Figure 1C). Furthermore, transfection of BHK cells with either human or Egyptian fruit bat ACE2 and subsequent infection with WIV1-CoV resulted in virus replication as measured in supernatant at 48 h post infection (hpi), whereas no virus replication could be found in BHK cells not transfected with ACE2 (Figure 1D). Taken together, these data show that WIV1 spike can effectively utilize ACE2 from Egyptian fruit bats for entry and infection.

**Figure 2:**
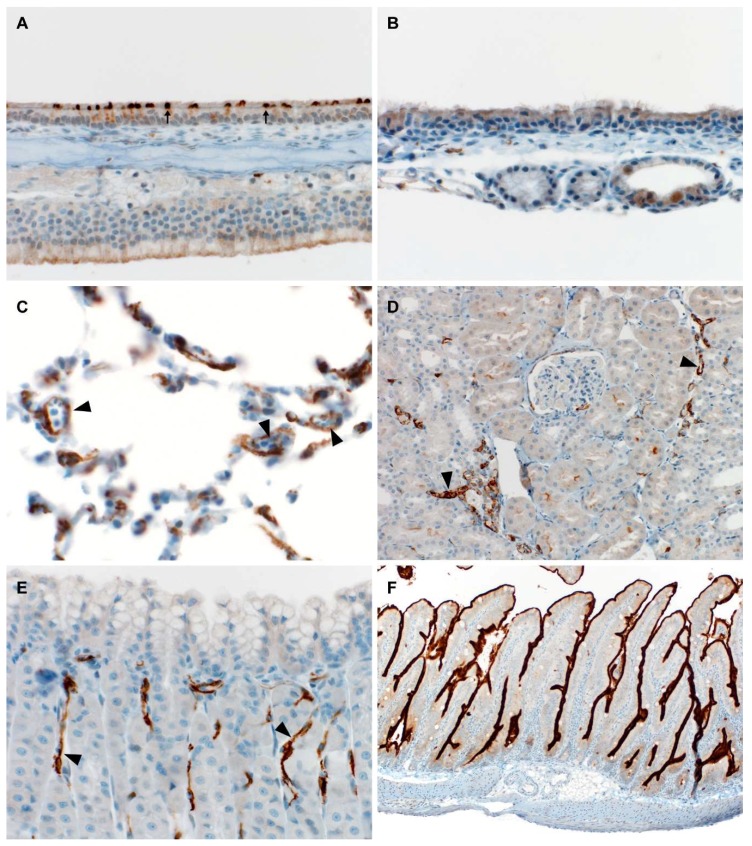
ACE2 immunohistochemistry of (**A**) nasal turbinate—400×, multifocal apical immunoreactivity in ciliated epithelial cells (arrows); (**B**) trachea—400×, negative immunostaining; (**C**) lung—400×, multifocal cytoplasmic endothelial cell immunoreactivity (arrowheads): (**D**) kidney—200×, multifocal cytoplasmic endothelial cell immunoreactivity (arrowheads); (**E**) stomach—400×, multifocal cytoplasmic endothelial cell immunoreactivity (arrowheads): (**F**) intestine—100×, diffuse immunoreactivity of brush border.

To confirm ACE2 expression in the organs of the Egyptian fruit bats, specific staining for ACE2 was performed on nasal turbinates, trachea, lung, kidney, stomach, and intestinal tissues. ACE2 expression was identified multifocally within the nasal turbinates, apical olfactory epithelium, serous submucosal glands, and pars nervosa of the pituitary gland. Cytoplasmic endothelial cell immunoreactivity was found in lung and stomach tissue. The kidney showed expression multifocally within interstitial capillaries and parietal epithelium. Intestinal tissue showed a bright diffuse immunoreactivity at the brush border (Figure 2). Together, the staining suggests necessary receptor expression to produce infection with WIV1-CoV in Egyptian fruit bats.

**Figure 3:**
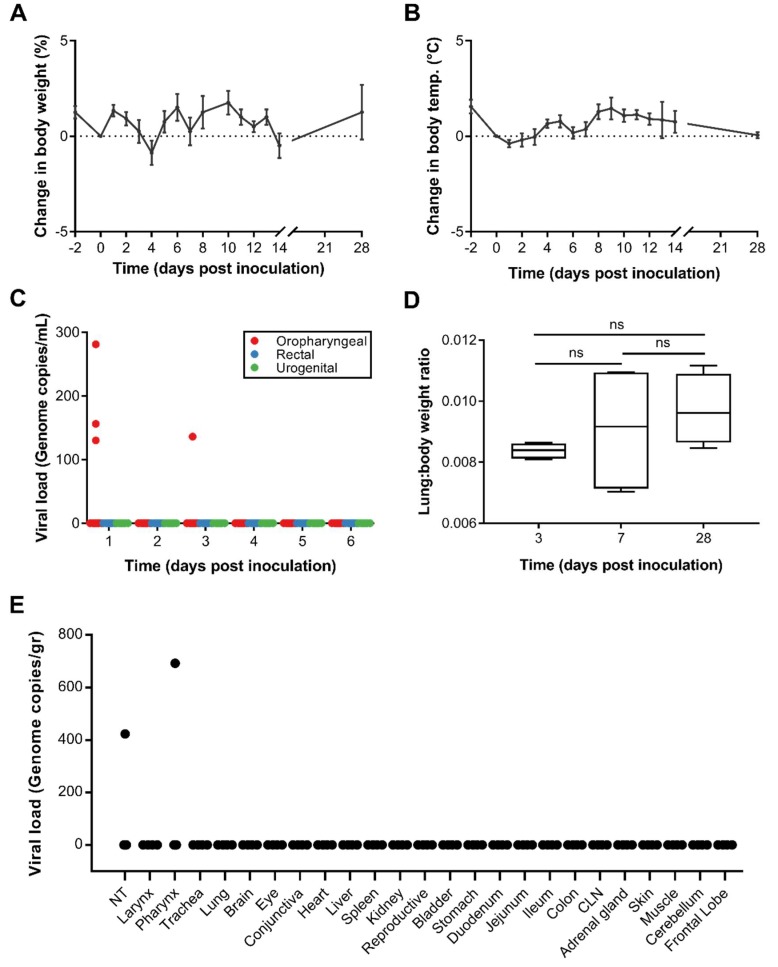
Disease symptoms, shedding and tissue tropism after inoculation with WIV1-CoV. (**A**) Relative weight change and (**B**) body temperature change in Egyptian fruit bats following inoculation with WIV1-CoV. (**C**) Viral RNA in oropharyngeal, rectal, and urogenital swabs obtained daily. (**D**) Lung:body weight ratio of Egyptian fruit bats at 3, 7, and 28 dpi. (**E**) Viral RNA load in tissues obtained 3 dpi (*N* = 4 bats).

Twelve Egyptian fruit bats were inoculated via the intranasal, intratracheal and intra-esophageal route with 10^5^ TCID_50_ of WIV1-CoV. These bats were observed daily for 14 days for any clinical signs. Their body weight and temperature were measured every day. None of the bats showed signs of disease such as respiratory distress, anorexia or lethargy. No significant body weight loss or changes in body temperature were detected throughout the experiment (Figure 3A,B).

Rectal, urogenital and oropharyngeal swabs were taken daily for six days and tested for the presence of viral RNA. Viral RNA could not be detected in rectal or urogenital swabs at any time point. However, on days 1 and 3 post inoculation (dpi) viral RNA was detected in the oropharyngeal swabs of three bats and one bat, respectively (between 100–300 copies/mL, Figure 3C).

Four bats each were necropsied on 3, 7, and 28 dpi. No postmortem gross pathological lesions were identified during necropsy. For each bat, the weight of the lungs was measured, and lung/body weight ratios were calculated. When comparing the lung/body weight ratios between animals taken on different days, no significant differences could be found (Student’s *t*-test, *p* ≥ 0.05, Figure 3D).

Multiple organ systems were examined histopathologically and were essentially normal with a few exceptions. All 12 bats had marked severe vacuolar change (glycogen-type) within the liver. The cause of this liver change was not identified but may be related to diet or stress. Six of the bats had minimal–moderate numbers of Kupffer cells in the liver containing a brown granular intracytoplasmic pigment predominately in the portal areas which are positive for Prussian Blue staining and consistent with excessive iron deposition. Seven bats had minimal–moderate numbers of lymphohistiocytic nodules within the pulmonary parenchyma. None of these histopathological changes were associated with coronavirus replication or immunohistochemistry suggesting that this was background pathology.

RNA was extracted from harvested tissues and analyzed for the presence of the host and viral RNA. Host RNA could be detected in all samples. Viral RNA was only detected in tissues obtained from animals necropsied on 3 dpi and was limited to the pharynx (*N* = 1) and nasal turbinates (*N* = 1) (Figure 3E). Subsequently, the full skull, as well as cervical lymph nodes, lung, spleen, and liver tissue, were stained for WIV1-CoV antigen using a SARS NP-specific antibody. All tissues were negative as determined by immunohistochemistry.

**Figure 4:**
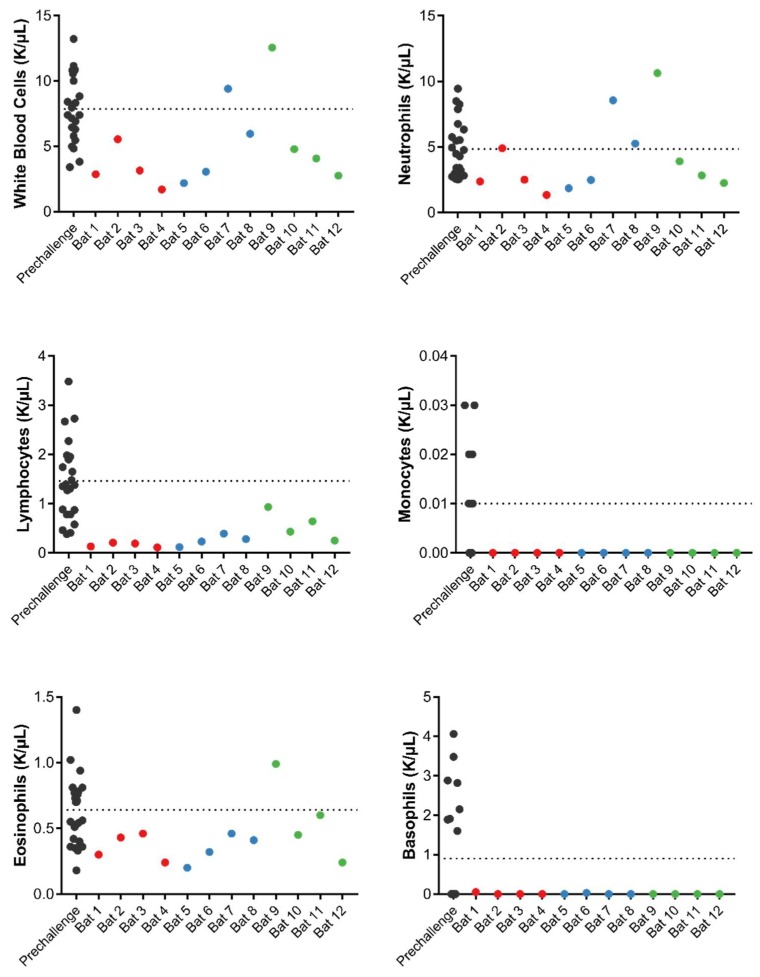
Hematology of Egyptian fruit bats inoculated with WIV1-CoV. Pre-challenge values (black) were obtained at D-95 (*N* = 12) and D-2 (*N* = 11). Post-challenge values were obtained at 3 dpi (red), 7 dpi (blue), and 28 dpi (green). All values were measured using the IDEXX ProCyte DX Analyzer. Dotted line = average of pre-challenge values.

Complete blood cell counts of four bats on each day of necropsy were compared to values obtained pre-challenge (two values per bat, 23 values in total). No abnormal values were detected in the levels of white blood cells, neutrophils, lymphocytes, monocytes, eosinophils or basophils (Figure 4). We hypothesize that the observed lymphopenia is stress-related, and not associated with viral infection.

**Figure 5:**
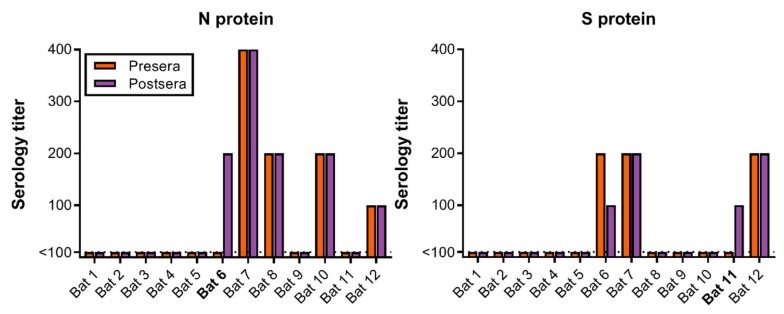
Serology titers in sera obtained pre- and post-challenge. ELISA assays were performed using SARS-CoV proteins N and S. Bats with an increase in serology titer are shown in bold.

The presence of antibodies was investigated using an ELISA, based on SARS-CoV S or N protein, and expressed as the highest dilution of sera that resulted in an OD > 0.2. Interestingly, sera obtained before challenge from several bats showed reactivity with SARS S or N (Figure 5). We compared the ELISA titer in sera obtained before and after challenge. Only two bats (bat #6 and #11) showed a significant increase in ELISA titer. No WIV1-CoV-neutralizing antibodies were detected in the sera as determined via virus neutralization assay. 

## 4. Discussion

In the present study, we investigated whether Egyptian fruit bats (*Rousettus aegyptiacus*) are susceptible to infection by SARS-like WIV1-CoV, originally detected in Chinese rufous horseshoe bats. The first step in host susceptibility to coronaviruses is associated with the ability to bind to host-specific receptors and the availability of these receptors on target organs. SARS-CoV S1 binds less efficiently to murine ACE2 than human ACE2, and thus wildtype mice are a suboptimal SARS-CoV animal model [10]. Upon expression of Egyptian fruit bat ACE2 on non-susceptible BHK cells, WIV1-CoV was able to enter and replicate, demonstrating that ACE2 from Egyptian fruit bats is a suitable receptor. However, while ACE2 was expressed in bat intestine and respiratory tract tissue, similar to humans [11], we observed very limited evidence of virus replication and seroconversion. Taken together, these data demonstrate that WIV1-CoV restriction in the Egyptian fruit bat was not at the receptor level. However, in this study very limited evidence of virus replication or seroconversion was detected. It is currently unclear why WIV1-CoV seems unable to replicate efficiently in Egyptian fruit bats. It is possible that the WIV1-CoV S glycoprotein is not processed by surface or intracellular proteases, which have been shown to be important host restriction factors during coronavirus entry [12,13]. Furthermore, a specific host factor may be required for virus transcription and replication, which could be lacking in the Egyptian fruit bats [14]. A recent study generated an annotated full genome for Egyptian fruit bats and used this to show greatly expanded natural killer cell receptors, MHC class I genes and type I interferons. It has been hypothesized that these factors allow greater tolerance to viral infection [15], resulting in prolonged infection but limited inflammation which has been described for Egyptian fruit bats inoculated with Marburg virus [16,17]. However, as we found very limited evidence of viral RNA, it is more likely that either an enhanced antiviral response or an inability of WIV1-CoV to dampen the innate response abrogated viral replication completely. Interestingly, some of the sera collected from bats before challenge contained antibodies which cross-reacted against full-length SARS-CoV spike protein as well as nucleoprotein. As most bats were negative for SARS-specific antibodies, we were unable to detect neutralizing antibodies. This does not directly explain why we did not see extensive viral replication. A small increase in the presence of SARS-CoV-specific antibodies was observed in 2 out of 12 bats; bat #6 showed an increase in N-specific antibodies, bat #11 showed an increase in S-specific antibodies. These findings suggest that some low-level virus replication might have occurred in these bats, resulting in an adaptive immune response.

We observed cleavage of MERS S glycoprotein in our pseudotypes, whereas neither SARS S or WIV1 S glycoprotein were cleaved. This matches closely to what has been reported on proteolytic activation of MERS S and SARS S glycoprotein and further supports the validity of the use of pseudotypes. Whereas MERS S glycoprotein is processed by host proprotein convertases in virus-producing cells before virus-cell entry [18], SARS S glycoprotein processing by these convertases is absent or inefficient [19]. Our results suggest that like SARS S glycoprotein, WIV1 S glycoprotein does not get cleaved by host proprotein convertases.

The obtained results suggest that the ability to infect and replicate efficiently is bat species-specific, and limited inferences can be made from non-matched host-pathogen systems. This has been observed with filoviruses as well. Marburg virus replicates efficiently in Egyptian fruit bats, which are the natural reservoir. However, other filoviruses, such as Ebola virus, hardly replicate in the Egyptian fruit bats [16]. Therefore, it would be interesting to perform an experimental inoculation study using Chinese horseshoe bats, from which WIV1-CoV was originally isolated, to investigate if more efficient virus replication and shedding can be observed in these animals.

In conclusion, we were unable to detect efficient replication of WIV1-CoV in Egyptian fruit bats and observed modest seroconversion in 2 out of 12 bats. Our findings add to the current understanding of the intricate relationship between specific bat species and their coronaviruses.
